# Foodborne Botulism in the United States, 1990–2000

**DOI:** 10.3201/eid1009.030745

**Published:** 2004-09

**Authors:** Jeremy Sobel, Nicole Tucker, Alana Sulka, Joseph McLaughlin, Susan Maslanka

**Affiliations:** * Centers for Disease Control and Prevention, Atlanta, Georgia, USA;; † Alaska State Department of Health and Social Services, Anchorage, Alaska, USA

**Keywords:** botulism, surveillance, epidemiology, foodborne disease, Clostridium botulinum, research

## Abstract

Foodborne botulism, a potentially lethal neuroparalytic disease, is caused by ingesting preformed *Clostridium botulinum* neurotoxin. We reviewed surveillance data and reports from 1990 to 2000. Of 263 cases from 160 foodborne botulism events (episode of one or more related cases) in the United States, 103 (39%) cases and 58 events occurred in Alaska. Patients' median age was 48 years; 154 (59%) were female; the case-fatality rate was 4%. The median number of cases per event was 1 (range 1–17). Toxin type A caused 51% of all cases; toxin type E caused 90% of Alaska cases. A particular food was implicated in 126 (79%) events. In the lower 49 states, a noncommercial food item was implicated in 70 (91%) events, most commonly home-canned vegetables (44%). Two restaurant-associated outbreaks affected 25 persons. All Alaska cases were attributable to traditional Alaska Native foods. Botulism prevention efforts should be focused on those who preserve food at home, Alaska Natives, and restaurant workers.

## Abstract 2

Home-canned foods and Alaska Native foods are leading causes of U.S. foodborne botulism; botulism’s epidemic potential renders each case a public health emergency.

Botulism is a paralytic illness caused by neurotoxins of the anaerobic, spore-forming bacterium, *Clostridium botulinum*, and rarely, by botulinum toxin–producing strains of *C. baratii* and *C. butyricum*. Seven immunologically distinct toxins exist, designated A through G. Types A, B, and E cause most human cases; type F cases have been reported rarely. Botulinum neurotoxins induce blockage of voluntary motor and autonomic cholinergic neuromuscular junctions, which prevents motor fiber stimulation. Clinical illness is characterized by cranial nerve palsies, followed by descending flaccid muscle paralysis, which can involve the muscles of respiration. Although ptosis and dysarthria may be mistaken for signs of encephalopathy, patients are fully alert, and the results of a sensory examination are normal. Recovery often takes weeks to months (*1*). The mainstays of therapy are meticulous intensive care unit support, with mechanical ventilation if needed, and administration of equine antitoxin (*2*). Timely antitoxin administration may arrest the progression of paralysis and decrease the duration of illness (*2*).

Foodborne botulism is a rare illness caused by eating foods contaminated with botulinum toxin. Spores of *C. botulinum* are ubiquitous in the environment (*3*), but growth and elaboration of toxin occur only under particular conditions that include an anaerobic, low-salt, low-acid environment. Bacterial growth is inhibited by refrigeration below 4°C, heating above 121°C, high water activity,or acidity (pH <4.5) (*4*). Toxin is destroyed by heating to 85°C for at least 5 minutes, and spores are inactivated by heating to 121°C under pressure of 15–20 lb/in^2^ for at least 20 minutes (*5*).

The canning and fermentation of foods are particularly conducive to creating anaerobic conditions that allow *C. botulinum* spores to germinate. Botulism was first described in consumers of sausages in Europe in the 18th century, and commercially canned foods caused outbreaks in the 19th and early 20th centuries before standard methods for inactivating *C. botulinum* spores in cans were perfected (*6*). Early in the 20th century, the proportion of botulism outbreaks caused by contaminated, commercially produced foods declined; however, improperly made home-canned foods have long constituted a major source of botulism in the continental United States (*1**,**7*). Since the 1970s, restaurant-associated botulism outbreaks have accounted for a large proportion of U.S. cases (*8*). Traditional Alaska Native foods, especially fermented foods like fish and fish eggs, seal, beaver, and whale, also pose a risk and account for the high incidence of botulism in Alaska (*9*). These foods, prepared by allowing the products to ferment at ambient temperatures, are often eaten without cooking.

Because contamination of a widely distributed food product could affect large numbers of persons, intensive surveillance is maintained for botulism cases in the United States, and every case is treated as a public health emergency. The Centers for Disease Control and Prevention (CDC) is the only source of therapeutic antitoxin, which is stocked in locations around the country for rapid release. CDC maintains a 24-hour clinical consultation and emergency antitoxin release service, and state health departments conduct epidemiologic investigations of suspected cases (http://www.cdc.gov/ncidod/dbmd/diseaseinfo/botulism.pdf) (*1*). Clinicians who suspect that they are treating a case of botulism should immediately contact their state health department's emergency telephone number. Botulinum toxin has been developed as a biologic weapon by various countries and terrorist groups and could be disseminated by deliberate contamination of foods or aerosolization (*10*). This situation adds urgency to early recognition and reporting of botulism cases. We review surveillance data on cases and outbreaks of botulism in the United States from 1990 to 2000.

## Methods

Suspected and confirmed botulism cases are reported to CDC by state health departments. Surveillance data, published and unpublished case reports, and outbreak reports were reviewed. A case of foodborne botulism was defined as a compatible illness in a person whose serum, stool, or gastric secretions tested positive for botulinum toxin; a compatible clinical illness in a person who ate food that tested positive for botulinum toxin; a compatible illness in a person whose stool was culture-positive for *C. botulinum*; a compatible illness in a person who ate food that was also eaten by a person with a laboratory-confirmed case; or a designation of the illness as foodborne botulism by the reporting state health department. An outbreak was defined as two or more cases of botulism caused by consuming a common source–contaminated food. An event was defined as the occurrence of a sporadic case or an outbreak of botulism. A food was considered the source of an outbreak if it tested positive for botulinum toxin and was eaten by a case-patient, or if an epidemiologic investigation linked a food to a botulism event, if the food was not tested. States other than Alaska were designated as the "contiguous states and Hawaii." Limited clinical data were collected at the time clinicians sought botulinum antitoxin from state health departments and CDC, and changes in data collection practices occurred during the period reported. Toxin detection and culture for *C. botulinum* were performed at CDC and 19 state and municipal public health laboratories according to standard methods (*5*). Limited clinical descriptors and outcome data were collected. Statistical analysis was performed using SAS version 8.2. (SAS, Cary, NC).

## Results

### United States

From 1990 to 2000, 160 foodborne botulism events affected 263 people in the United States, an annual incidence of 0.1 per million. No discernable trend was evident in the overall or toxin type–specific annual case counts (Figure). The median number of cases per year was 23 (range 17–43), the median number of events per year was 14 (range 9–24), and the median number of cases per event was 1 (range 1–17). The highest incidence rates were in Alaska, Idaho, and Washington (Table 1). One hundred and thirty-one cases (50%) were caused by toxin type A, 27 (10%) by toxin type B, and 97 (37%) by toxin type E (Table 2). Among case-patients for whom data were available, 67 (26%) persons were intubated. Forty-one (84%) persons with type A intoxication were intubated, compared with 6 (33%) persons with type B and 17 (33%) of those with type E. For case-patients with available data, 7 persons (5%) with type A intoxication died, compared with 1 (4%) with type B and 3 (3%) with type E.

**Figure:**
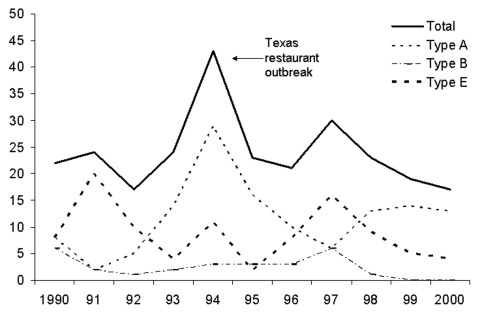
Foodborne botulism cases in the United States, 1990–2000.

**Table 1:** States with five or more foodborne botulism cases, 1990–2000

State	No. cases	Incidence/1,000,000^a^
Alaska	103	19
California	37	0.1
Texas	20	0.1
Washington	18	0.3
New Jersey	10	0.1
Georgia	8	0.1
Idaho	7	0.6
Oregon	6	0.2
Ohio	5	<0.1

^a^Using intercensal estimate, U.S. Census Bureau data.

**Table 2:** Toxin types of foodborne botulism cases and events in the United States, 1990–2000

Toxin type	No. cases (%)	No. events (%)
A	130 (50)	80 (50)
B	27 (10)	23 (14)
E	97 (37)	52 (33)
F	3 (1)	3 (1)
Unknown	6 (2)	5 (2)
Total	263 (100)	160 (100)

### Contiguous States and Hawaii

#### Events

In the contiguous states and Hawaii, 102 foodborne botulism events occurred, which affected 160 persons; a median of 9 events (range 4–13) and 14 cases (range 4–30) took place per year. The median number of cases per event was 1 (range 1–17). The median age of case-patients in the contiguous states was 50 years (range 4–88 years), and 83 (52%) were female. The overall case-fatality rate was 5%. No clear seasonal pattern was observed.

A food was implicated by laboratory detection of toxin or epidemiologic investigation without laboratory confirmation in 77 (76%) events (Table 3). Of these events, 68 (67%) were caused by homemade foods. Home-canned foods accounted for 47 (69%) of the homemade food events, affecting 70 people, while other types of homemade foods accounted for the remaining 21 (31%) events, which affected 27 people. Of the nine events caused by nonhomemade foods, five (56%) events, which affected 10 people, were caused by commercial foods, and two (22%) events, which affected 25 people, were caused by restaurant-prepared foods.

**Table 3:** Foodborne botulism events, United States, 1990–2000^a^

Type of processing/food	No. events^b^	No. cases
Noncommercial, home canned		
Asparagus	9	14
Squash	1	2
Peppers	2	4
Corn	1	1
Beans	2	3
Pumpkin	1	1
Greens	2	2
Tomato juice	1	1
Olives	4	4
Beets	3	6
Not specified	3	4
Vegetables, no further specificity	3	3
Mushrooms	2	2
Soup, no further specificity	1	2
Stew, no further specificity	1	3
Potatoes	1	1
Tuna	2	4
Eggplant	1	2
Turnips	1	1
Carrots	1	3
Eggs	1	1
Chiles	1	1
Pickles	1	1
Garlic in oil	2	4
Total	47	70
Noncommercial, not home canned		
Sausage	2	3
Salsa	2	2
Potato salad	2	3
Bread pudding	1	1
Liver paté	1	1
Soup, no further specificity	4	4
Beef chili	1	2
Meatballs and sauce	1	1
Roast beef	1	1
Apple pie	1	1
Potatoes	3	3
Hamburger	1	1
Pickled herring	1	4
Total	21	27
Commercial		
Salted,uneviscerated fish (mohola)	1	3
Palani (surgeon fish)	1	3
Burrito	1	1
Clam chowder	1	2
Bean dip	1	1
Total	5	10
Restaurant-made		
Cheese sauce	1	8
Skordalia potato dip	1	17
Total	2	25
Other		
Peyote tea	1	1
Unknown	25	26
Total, contiguous states and Hawaii	102	160
**Alaska**		
Noncommercial		
Seal oil	14	20
Fish eggs	7	18
Fermented sea mammals	11	21
Fermented fish	14	28
Mixed ingredients	3	5
Total	49	92
Unknown	913	11
Total, Alaska	58	103
**Total, United States**	160	263

^a^Foods were implicated either by isolation of toxin from the food or by epidemiologic investigation without laboratory confirmation. ^b^Event was defined as a sporadic case or outbreak of >2 related cases.

#### Improper Food Handling Practices

Improper food handling practices that permit germination and growth of *C. botulinum* with subsequent toxin elaboration were identified in events involving noncanned homemade foods. For example, salsa made with raw vegetables that were placed in nonrefrigerated airtight plastic containers, which likely fostered an anaerobic environment, was associated with events in 1990 and 1993; each event involved one case-patient. Home-bottled garlic-in-oil was associated with events in 1991 in California and in 1999 in Florida. In one of these events, the garlic-in-oil was prepared by using home-canning methods; the mixture was heated to a temperature insufficient to kill *C. botulinum* spores (*11*). Lastly, investigators have suggested that failure to refrigerate freshly made home-cooked foods not normally associated with botulism, such as the beef chili implicated in an outbreak affecting two persons in New Jersey in 2000, can result in toxin production and illness.

Improper food handling practices identified in the five events caused by commercially produced foods (Table 3) include the following. First, an outbreak of botulism type E, affecting three people, was caused by mohola, a salt-cured traditional Egyptian fish product, reportedly purchased at an ethnic New Jersey retail fish market in 1992 (*12*). Second, an event of botulism type B, which affected one person in Oregon in 1997, was caused by a commercially produced burrito purchased at a roadside store. Third, an event of botulism type A, which affected one person in California in 1994, was attributable to a commercially produced bean dip that was stored in an airtight plastic container at room temperature; the "keep refrigerated" instruction on the container was in minuscule print. Fourth, an outbreak of botulism type A, which affected two persons in California in 1994, was caused by a commercially produced clam chowder. The chowder was a low-acid product packed in a sealed plastic bag. Though the product was perishable and was sold in the refrigerated section of a grocery store, it carried no "perishable" label with instructions to refrigerate, as required by California state law. The case-patients stored the soup at room temperature for several weeks and then ate it despite its bad odor and flavor. Fifth, an outbreak of botulism type B, affecting three persons in Hawaii in 1990, was caused by commercially caught, retail-sold palani (surgeon fish). The fish was bought fresh, grilled, and eaten immediately; severe illness appeared to be associated with eating the cooked fish intestines (*13*).

Two outbreaks caused by restaurant-prepared foods involved skordalia, a potato-based dip, and a commercially canned cheese sauce. The first outbreak occurred in Texas in 1993 and affected 17 persons. The skordalia was prepared with potatoes that were baked in aluminum foil wrapping, then left wrapped in the foil at ambient temperature for several days (*14*). The second outbreak occurred in Georgia in 1993 and affected eight persons. It involved canned cheese sauce eaten after the opened can was left in the restaurant without refrigeration (*15*).

#### Botulinum Toxin Types

During the period under study, 60 events were caused by type A botulinum toxin. The implicated foods in 40 (50%) were home-canned products (27 vegetable items, two home-canned soups, two home-canned tuna items, one each home-canned tomato juice, garlic-in-oil, and stew). Other implicated home-prepared foods included five events from potato salad or potatoes, four from homemade soup, two from homemade sausage, and one from each of the following: roast beef, liver paté, bread pudding, salsa, apple pie, hamburger, and chili. In 18 (23%) events caused by botulinum toxin type A, the specific food vehicle was not identified.

During the period under study, 15 events were caused by type B botulinum toxin. The implicated foods in five (33%) of the type B toxin events were home-canned products (corn, eggs, green beans, olives, and an unspecified vegetable), and one event was due to each of the following: a commercially manufactured burrito, commercially caught and sold fish, pasta sauce and meatballs, salsa, turnip greens, and peyote. In four (25%) events, the food vehicle was not identified.

Three events, all attributable to consumption of fish, were caused by botulinum toxin type E. In one event, the toxin type was not identified.

### Alaska

During the period under study, 58 botulism events occurred in Alaska; 103 persons were affected, with a median of 5 events (range 0–15) and 8 cases (range 0–20) occurring per year. The median number of cases per event was 1 (range 1–5). Most events occurred in the spring through the fall, with a sharp peak in July. The median age of case-patients was 44 years (range 8–92), and 70 (68%) were female. The case-fatality rate was 3%. The contaminated food was identified in 49 (84%) of events. All identified foods were homemade Alaska Native foods. Eleven events, affecting 21 persons, were caused by foods consisting of fermented aquatic mammal tissues, such as muktuk (whale skin and blubber), beaver tail, and seal flipper. Fourteen events, affecting 20 persons, were caused by seal oil; 14 events, affecting 28 persons, were caused by fish foods such as fermented salmon heads and whitefish; 7 events, affecting 18 persons, were caused by fermented fish eggs; and 3 events, affecting 5 persons, were caused by foods with mixed ingredients. Forty-nine (84%) events and 91 (88%) cases were caused by toxin type E, all involving foods from aquatic animals. Eight (14%) events with 11 (11%) cases were caused by toxin type B; 6 of these events were caused by animal foods of aquatic origin, and two were caused by unknown foods. In one event caused by fermented seal, the toxin type was not identified.

#### U.S. Foodborne Botulism Cases with No Food Vehicle Identified

During the period under study, 37 cases of botulism were reported by state health departments as foodborne botulism attributable to an unknown food; these represented 14% of all cases. Of these, six were associated with outbreaks of two or more cases and therefore can be considered with certainty to be foodborne. The characteristics of the remaining 31 sporadic cases reported as foodborne, but not having an identified food, resembled those of other foodborne botulism cases. Twenty-two (61%) patients were female, and the median age was 55 (range 42–84). Seventeen (55%) cases were caused by toxin type A, 6 (19%) by toxin type B, 5 (16%) by toxin type E, and 3 (10%) by toxin type F. One (3%) case-patient died.

## Discussion

In previous eras, conditions conducive to the survival of *C. botulinum* spores and their subsequent germination in food were much more common. Nevertheless, enduring methods of preparing certain homemade foods, new ways of packaging commercial foods, new food preferences, or new techniques for preparing familiar foods that support the growth of *C. botulinum* render it likely that foodborne botulism will afflict humans for the foreseeable future.

From 1990 to 2000, home-canned foods remained a leading cause of foodborne botulism in the United States. New interventions should be explored to ensure that methods of home canning vegetables incorporate adequate barriers to prevent *C. botulinum* germination. Possible areas of research may include development of practical dye indicators for pH and temperatures above those of refrigeration. Botulism associated with Alaska Native foods is likely an age-old problem, compounded in recent decades by altering traditional practices in an unsafe manner, in particular, to include the use of plastic or glass containers for fermentation. To address this problem, the Alaska State Department of Health and Social Services, in partnership with CDC, has developed culturally appropriate educational materials on safer native food preparation. The preventive message focuses on handwashing, using safer, traditional fermentation processes, avoiding plastic and glass containers, shielding fermenting food from sunlight, boiling native foods before consumption, and discarding suspicious foods (http://www.phppo.cdc.gov/phtn/botulism/default/default.asp) (*16*).

*C. botulinum* rarely causes illness because the confluence of conditions required for its germination and toxin production—low acidity, high water activity, absence of preservatives, ambient temperature, and anaerobic milieu—rarely occurs in foods (*4*). However, from 1990 to 2000, preservative-free, low-salt, commercially produced foods packaged in air-tight containers with no intrinsic barriers to *C. botulinum* spore germination were popular, possibly because they are perceived by the public as healthier. The sole barrier to *C. botulinum* germination in these foods is refrigeration. The outbreaks attributable to commercial bean dip and clam chowder exemplify the failure of this lone safeguard. Prevention could be enhanced by mandating multiple barriers to spore germination, such as acidification and reduced water activity, along with prominent refrigeration instructions, in all low-oxygen packaged commercial foods.

Botulism from traditional recipes of uneviscerated fish is well documented in such preparations as Egyptian fasikh (*17*) and East-European kapchunka (*18*). The 1992 outbreak from moloha (Table 3) falls into this category. Current regulations prohibit the sale of uneviscerated fish (*19*). The outbreak from palani in Hawaii in 1990 is novel because the commercially caught fish was purchased fresh at a retail establishment, cooked, and served immediately (*13*). Control measures include appropriate refrigeration between catch and sale, similar to those used to control scombroid poisoning (*20*). From 1990 to 2000, restaurant-associated outbreaks continued to cause a disproportionate number of botulism cases. Investigation of the skordalia dip outbreak identified the practice of leaving baked potatoes wrapped in foil at ambient temperature as the principal hazard; this practice must be completely eliminated from restaurants and homes. Control measures depend on the education of restaurant operators and enforcement by sanitation authorities.

Thirty-one reported sporadic foodborne cases had no identified contaminated food source. Were these, in fact, cases of foodborne botulism? The median age, gender distribution, and fatality rate were similar to those of other foodborne cases, and no patients were reported to be injection drug users, the leading risk factor for wound botulism (*21*). However, some of these may represent cases of adult intestinal toxemia botulism (AITB) or adult-infant botulism. The diagnosis of AITB is difficult to establish because it requires proof of in vivo toxin production by demonstrating botulinum toxin or *C. botulinum* in patient specimens over time (*22*). Absence of a toxin-containing food, identification of *C. botulinum* in stool, and a history of abnormal bowel anatomy or recent antimicrobial drug use support the diagnosis of AITB (*23**,**24*). Some AITB cases may have been misclassified as foodborne. All three cases of botulism type F reported from 1990 to 2000 were reported as foodborne but with no implicated food. Since identification of type F toxin in food is practicable (*25**,**26*), failure to implicate a food may indicate these cases were misclassified AITB. For patients with sporadic cases of adult botulism with no implicated food or injection drug history, repeated stool cultures over time may help establish the diagnosis of AITB. As the clinical syndrome of inhalational botulism probably does not differ from that of foodborne botulism (*27*), the possibility of aerosol exposure should be considered in otherwise unexplained cases.

Our data have several limitations. Most clinical data were collected at one point, when antitoxin was sought by the treating clinician from CDC or a state health department. Changes in reporting format during the period reported resulted in incomplete data for a substantial portion of cases. Differences in toxin type–specific rates of intubation and deaths on a subset of cases must be cautiously interpreted in light of these facts.

Foodborne botulism, while rare, remains a public health emergency because of its severity and epidemic potential. Home-canned foods and Alaska Native foods remain the leading causes in the United States, and restaurant-associated outbreaks continue to account for a disproportionate number of illnesses. All suspected cases of botulism should be reported to public health authorities immediately. Prompt epidemiologic investigation helps prevent additional cases and can identify new risk factors for intoxication.
